# Emerging Priorities for Microbiome Research

**DOI:** 10.3389/fmicb.2020.00136

**Published:** 2020-02-19

**Authors:** Chad M. Cullen, Kawalpreet K. Aneja, Sinem Beyhan, Clara E. Cho, Stephen Woloszynek, Matteo Convertino, Sophie J. McCoy, Yanyan Zhang, Matthew Z. Anderson, David Alvarez-Ponce, Ekaterina Smirnova, Lisa Karstens, Pieter C. Dorrestein, Hongzhe Li, Ananya Sen Gupta, Kevin Cheung, Jennifer Gloeckner Powers, Zhengqiao Zhao, Gail L. Rosen

**Affiliations:** ^1^School of Biomedical Engineering, Science and Health Systems, Drexel University, Philadelphia, PA, United States; ^2^The School District of Philadelphia, Philadelphia, PA, United States; ^3^Department of Infectious Diseases, J. Craig Venter Institute, La Jolla, CA, United States; ^4^Department of Nutrition, Dietetics and Food Sciences, Utah State University, Logan, UT, United States; ^5^Ecological and Evolutionary Signal-processing and Informatics Laboratory (EESI), Electrical and Computer Engineering, Drexel University, Philadelphia, PA, United States; ^6^College of Medicine, Drexel University, Philadelphia, PA, United States; ^7^Nexus Group, Faculty of Information Science and Technology, Gi-CoRE Station for Big Data & Cybersecurity, Hokkaido University, Sapporo, Japan; ^8^Department of Biological Science, Florida State University, Tallahassee, FL, United States; ^9^Department of Civil Engineering, New Mexico State University, Las Cruces, NM, United States; ^10^Department of Microbiology, The Ohio State University, Columbus, OH, United States; ^11^Department of Microbial Infection and Immunity, The Ohio State University, Columbus, OH, United States; ^12^Department of Biology, University of Nevada, Reno, Reno, NV, United States; ^13^Department of Biostatistics, Virginia Commonwealth University, Richmond, VA, United States; ^14^Department of Medical Informatics and Clinical Epidemiology, Oregon Health & Science University, Portland, OR, United States; ^15^Department of Obstetrics and Gynecology, Oregon Health & Science University, Portland, OR, United States; ^16^Collaborative Mass Spectrometry Innovation Center, Skaggs School of Pharmacy and Pharmaceutical Sciences, University of California, San Diego, San Diego, CA, United States; ^17^Department of Biostatistics, Epidemiology and Informatics, Perelman School of Medicine, University of Pennsylvania, Philadelphia, PA, United States; ^18^Department of Electrical and Computer Engineering, The University of Iowa, Iowa City, IA, United States; ^19^Department of Dermatology, The University of Iowa, Iowa City, IA, United States

**Keywords:** microbiome interactions, gut microbiome, skin microbiome, prebiotics, probiotics, microbiome evolution, microbiome ecology, microbial forensics

## Abstract

Microbiome research has increased dramatically in recent years, driven by advances in technology and significant reductions in the cost of analysis. Such research has unlocked a wealth of data, which has yielded tremendous insight into the nature of the microbial communities, including their interactions and effects, both within a host and in an external environment as part of an ecological community. Understanding the role of microbiota, including their dynamic interactions with their hosts and other microbes, can enable the engineering of new diagnostic techniques and interventional strategies that can be used in a diverse spectrum of fields, spanning from ecology and agriculture to medicine and from forensics to exobiology. From June 19–23 in 2017, the NIH and NSF jointly held an Innovation Lab on *Quantitative Approaches to Biomedical Data Science Challenges in our Understanding of the Microbiome*. This review is inspired by some of the topics that arose as priority areas from this unique, interactive workshop. The goal of this review is to summarize the Innovation Lab’s findings by introducing the reader to emerging challenges, exciting potential, and current directions in microbiome research. The review is broken into five key topic areas: (1) interactions between microbes and the human body, (2) evolution and ecology of microbes, including the role played by the environment and microbe-microbe interactions, (3) analytical and mathematical methods currently used in microbiome research, (4) leveraging knowledge of microbial composition and interactions to develop engineering solutions, and (5) interventional approaches and engineered microbiota that may be enabled by selectively altering microbial composition. As such, this review seeks to arm the reader with a broad understanding of the priorities and challenges in microbiome research today and provide inspiration for future investigation and multi-disciplinary collaboration.

## Introduction

Microbiome research, which focuses on the behavior, interactions, and function of microbial communities within a specified environment, has made tremendous gains over the past 15 years (McEnery, 2017). These advances have been driven in large part by the dramatic cost reduction of high-throughput screening and increase in computational power over this period, which has provided a flood of data that can be efficiently processed on ubiquitous hardware. From this data, our understanding of the human and environmental microbiomes has increased exponentially, and more discoveries continue to be made every day. Herein, we present a review of the current priorities in microbiome research and challenges at the frontiers of this rapidly accelerating field.

We start by discussing interactions between microbes and the human body and provide examples of current research on the physiological effects of such interactions within the body. We continue by identifying considerations that affect the evolution and ecology of microbes, including the role played by the environment and microbe-microbe interactions. Next, we introduce some of the most important analytical and mathematical methods used in current microbiome research. We then present a discussion about how the microbial composition may be used for diagnostics and classification and discuss exemplary applications. Finally, we conclude by identifying potential interventional approaches that may be enabled by selectively altering microbial communities.

## Host-Environment-Microbiome Interactions, Evolution, and Ecology

The microbiome, defined as a set of highly interactive microbial species, is shaped by the environment in which it exists, which includes hosts, and exogenous natural and human factors. In this section, we present a discussion on the microbiome and the role of microbe-environment interactions on the ecology and its evolution.

### Host-Microbe Interactions

The term “holobiont” (Margulis and Fester, 1991) refers to a host and all its associated microbes, and the term “hologenome” refers to the genomes of the host and the microbes. Some scientists consider the holobiont as the unit upon which natural selection acts, whereas others have criticized this metaphor, and question whether the microbiome can respond to natural selection, given its limited heritability (Moran and Sloan, 2015; Douglas and Werren, 2016; Davenport et al., 2017).

Despite important differences in the microbiome of different individuals of any given species (associated with diet, environment, etc.), these microbial communities usually vary less among individuals within a species than between different species. Each host species has a core microbiome comprised of microbial taxa that are present in most individuals and that likely carry out essential functions, and a peripheral microbiome made up of all other identified taxa, which probably carry out accessory functions.

Each host can acquire microbes in two ways: vertically (inheriting them from the parents) or horizontally (acquiring them from the environment, including food and other individuals of the same and other species). Vertical transmission results in the phylogeny of hosts correlating with microbiomes similarity (with closely related species exhibiting similar microbiomes), a pattern known as “phylosymbiosis” – it should be noted, however, that phylosymbiosis can emerge due to mechanisms other than vertical transmission, e.g., due to close contact with other members of the host species (Sanders et al., 2014; Groussin et al., 2017). Vertical transmission can also result in co-speciation (microbes speciating as a result of host speciation) and co-diversification (microbes exhibiting similarity evolutionary histories due to co-speciation or similar selective pressures) (Davenport et al., 2017). Horizontal transmission, on the other hand, tends to erode phylosymbiosis as it mixes evolutionary histories and breaks up these associations.

Multiple lines of evidence indicate that vertical transmission is a major force shaping microbiome evolution, both at the level of community composition and of individual bacterial cells. A phylogeny inferred from the gut microbiome composition of different great apes is perfectly congruent with the great ape phylogeny, despite frequent horizontal acquisition of new microbes (Ochman et al., 2010). In addition, individual bacterial lineages exhibit phylogenies that resemble that of great apes, both topologically and in their divergence times, thus indicating co-speciation and co-diversification (Moeller et al., 2016a). Germ-free mice can be colonized by gut bacteria from other species as distant as humans, but the success of the colonization and its beneficial effects on the host (bacteria are necessary to fully develop intestinal immunity) depend on how closely related the donor species is to mouse (Chung et al., 2012; Seedorf et al., 2014; Brooks et al., 2016).

### Host-Environment-Microbiome Interactions

#### Ecology and the Environment-Microbiome Nexus

Current microbiome research is highly biased toward aspects pertaining to human health (Waldor et al., 2015; Karkman et al., 2017; Martí et al., 2017). However, in the broader realm of ecosystem health, human health reflects a single dimension of interaction of the microbiota with the environment. The reality is that it is more and more accepted that an healthy environmental microbiome determines a healthy human microbiome (Lloyd-Price et al., 2016). Thus, it is critical to study ecosystems’ microbiome. The structure and functional richness of ecosystems’ communities at different scales of biological organization are important in determining the microbiome of individuals and populations (Dermyshi et al., 2017; Rees et al., 2017). Additionally, human populations profoundly influence the surrounding availability of environmental microbes in urbanized areas, creating non-linear feedback loops that are far from being understood (Pinto et al., 2014; Krause et al., 2017; Van Rossum et al., 2018). Multi-scale variability, universality of microbiome drivers, and geographical dependency (Bashan et al., 2016; Falony et al., 2016) are further topics yet to be investigated. Here, we first focus on the ecology of the microbiome and (i) how ecological theory can help in understanding microbiome dynamics in natural and human communities (Costello et al., 2012), (ii) how the environment-microbiome nexus is shaped between natural and human communities (Lynch and Neufeld, 2015), and (iii) how engineering strategies can try to control harmful effects related to microbiome alterations in ecosystems (see for example Bucci et al., 2012). This review does not address any individual microbiome variability, as population-level approaches can be considered more appropriate for understanding and controlling microbiome-related outcomes.

#### On the Ecology of the Microbiome in a Population

**FIGURE 1:**
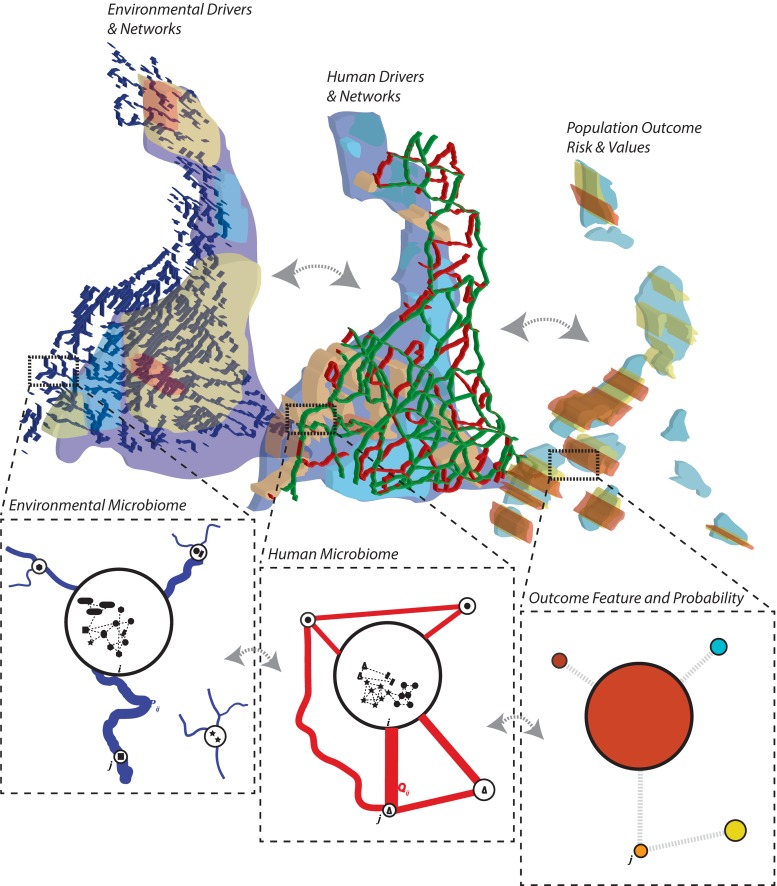
Metacommunity approach for studying the ecology and evolution of the microbiome. The ecosystem is discretized in communities (nodes) connected via environmental and human links representing relevant connection determining the spread of species and/or hosts such as river networks and human mobility networks (Convertino et al., 2009; Coyte et al., 2015; Bashan et al., 2016). Local/nodal environmental and human features constitute the likely niche of species to exist in a community. The human-environmental microbiome nexus (HEM), that is the multiplex network between functionally relevant microbiome networks in the human population and the environment, determines some population outcomes of interest (such as diseases in human populations, and other ecological outcomes such as collective population abundance and functional diversity in animal populations). Each node of the community can contain a detailed characterization of the microbiome interaction network or graph (see Figure 4). Systemic inter-community networks can also be inferred from information theoretic models (Convertino and Valverde, 2019; Li and Convertino, 2019) or statistical models based on interdependence of microbial patterns.

Community ecology is a discipline that is now deeply consolidated theoretically, empirically, analytically and computationally. In the context of a microbiome, diversity is a key factor in determining the stability of the microbiome and the microbiome-related health of a population (Costello et al., 2012; Coyte et al., 2015). Functional diversity rather than taxonomic diversity is a much more fundamental and meaningful feature highlighting the state of the microbiome (Li and Convertino, 2019). However, functional diversity is difficult to measure and taxonomic diversity, if properly accounting for collective endemic interspecies abundance distribution, can be meaningful of microbiome states and configurations (Woloszynek et al., 2019). In particular, here we focus on a metacommunity representation of the microbiome (Leibold et al., 2004; Convertino et al., 2009; Coyte et al., 2015) composed of multiple interacting communities, determining diversity within (alpha) and between (beta) communities or assemblages, as well as total diversity of microbial species (gamma diversity). These communities at different biological and spatio-temporal scales (Figure 1) shares information fluxes that are representative of microbial interdependencies. Leaving aside niche versus neutral theories of organization (Chao et al., 2005; Hubbell, 2006; Kraft et al., 2008), the metacommunity approach is well-developed and highly useful for predicting biodiversity assemblage and shifts, its stability, and determining local and systemic drivers of diversity, particularly in spatially defined communities (Leibold et al., 2004).

#### Human – Environment-Microbiome Nexus

The connection between environmental and human health dynamics in short and long time scales is a microbiome research area that has not been well tackled yet at the population scale. Recent efforts are mapping the microbiome of Earth for different habitat types (see for instance the Earth Microbiome project^1^ and the Global Ocean Microbiome project^2^), however, the connection between environment and population microbiome is still lacking and difficult to predict. While it is true that consistent efforts have been devoted to the analysis of disease- or symptom-specific alterations of the microbiome in relation to external environmental agents (Bucci et al., 2012; Davenport et al., 2017; Karkman et al., 2017; Mitmesser and Combs, 2017), a large gap exists in the analysis of how the spatio-temporal distribution of microbiota in the environment [e.g., soils (Jansson and Hofmockel, 2018), plants (Wackett, 2019), water (Lee et al., 2016), and natural hosts (Bahrndorff et al., 2016; Degli Esposti and Martinez Romero, 2017)] affects the microbiome in natural and human communities. Note that this ecological investigation, guided by theory, targeted monitoring and models (see section “Pattern-Oriented Models”), does not necessarily need to focus on health but on any spatio-temporal pattern manifesting ecological states of co-evolving microbiomes such as biodiversity patterns [see Parfrey and Knight (2012) and Ochman et al. (Moeller et al., 2016b; Ochman, 2016)] and other socio-ecological ecosystem services.

Within the metacommunity framework, some specific research questions are about to determine the extent of source-sink habitat dynamics for harmful or nuisance species, and the frequency with which each is sourced from human or environmental communities. For example, it is interesting to know whether harmful microbiota share intrinsic ecophysiological traits with traditional invasive species, such as offspring quality/quantity-selection and high dispersal abilities. It has been suggested that microbial biodiversity may boost immunity to bacterial infections (Lax et al., 2015), potentially by conferring resistance to invasion by new species, but this dynamics has considered only taxonomic diversity that has limited explanatory power when considering microbiome function.

In this perspective, the theory of multiplex networks (see Servadio and Convertino, 2018) and Li and Convertino (2019) for an example of these methods considering a portfolio of health outcomes and microbial species interactions biodiversity patterns) works well in representing co-evolving non-linear species, or metacommunities, subjected to stochastic environmental dynamics. Assumption-free pattern-oriented models developed in a metacommunity perspective can detect main local drivers of microbial diversity (Convertino et al., 2013), fundamental dispersal corridors (Martí et al., 2017), alternative stable and transitory states (Rees et al., 2017), stressor-dependent variability and resilient mechanisms associated to natural stationary conditions or specific population outcomes (Shade et al., 2012; Gonze et al., 2017; Zaneveld et al., 2017; Li and Convertino, 2019; Figure 1). Certainly, models are just a component in future microbiome research, but these data- and theory-based models should also guide field data collection and *in vitro* experiments (Widder et al., 2016) in order to have an optimal environment-microbiome nexus exploration.

#### Diet and Its Effect on Gut Microbiome Composition and Function

**FIGURE 2:**
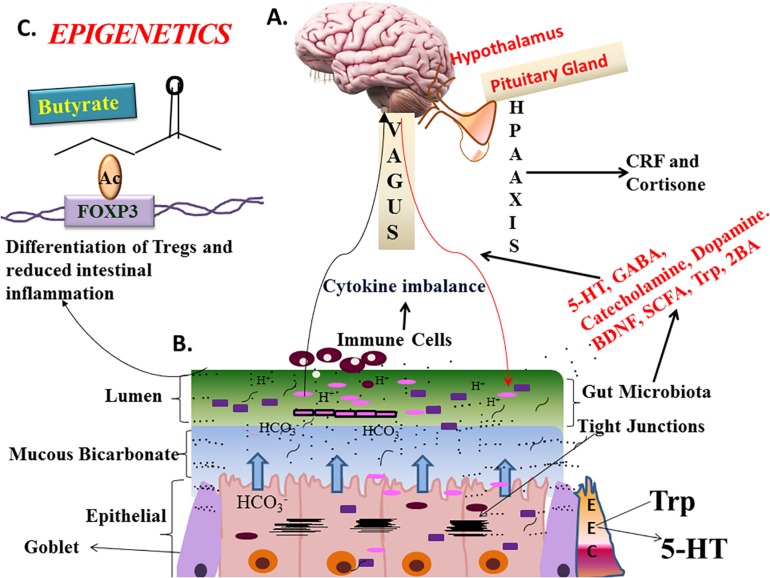
State-of the–science-Gut-Brain-Bidirectional Axis (GBM). Three ways microbes communicate with GBM: neurobiochemical, neuroendocrinal, and neuroimmune mechanisms. Microbial sps can modulate hypothalamus-pituitary-adrenal gland (HPA) axis, by affecting corticotrophin releasing factor (CRF), and cortisone levels which can subsequently affect intestinal permeability and cause hypersensitivity. Neuroactive molecules like γ-aminobutyric acid (GABA), 5-HT, norepinephrine, and dopamine are produced independently by bacteria or through digestion of other food sources. *Lactobacillus subspecies, Candida, Streptococcus, E. coli*, and *Enterococcus* can make 5HT which affects sleep, appetite, mood, and cognition (Liu and Zhu, 2018). *Clostridiales* regulate synthesis and release of 5-HT by making tryptophan available (Martin et al., 2018) for its synthesis. Vagus nerve is the major connection between microbiome and gut, is imperative for GBM-axis. Microbial metabolites like short chain fatty acids, bile acids, and tryptophan can communicate between gut and brain directly or through vagal/spinal highways. Stress, dietary changes and microbiome can lead to cytokines imbalance and increases the risk of intestinal inflammation, IBD, and allergies, etc. Gut microbiota made metabolites like butyrate have epigenetic effect on FOXP3 (forkhead box P3) promoter of T-regs (Furusawa et al., 2013). Prebiotics like fructo-oligosaccharides and galacto-oligosaccharides increase BDNF, serotonin, GABAb receptor levels while reducing cortisone and L-Trp, hence have anti-anxiety and anti-depressant effect. Prebiotics and probiotics regulate the capacity of intestinal microbiota, preserve the integrity of the intestinal barrier (enteroendocrine cells), prevent bacterial translocation and regulate local inflammatory reaction through the intestinal related immune system. BDNF, Brain-derived Neurotrophic Factor; 5-HT, Serotonin; Trp, Tryptophan; SCFA, Short-chain fatty acid. **(A)** Showing human brain with detailed picture of hypothalamus and pituitary gland **(B)**. Showing the gastric mucosa lined by epithelial, goblet, and enterochromaffin cells (EEC), gastric mucosa is also showing bicarbonate buffer and lumen which has most of the microbiota (from Wikipedia) **(C)**. Showing the epigenetic effect of butyrate on FOXP3 promoter.

Most of microbiome research focuses on coexisting microbes and host-microbe interactions. One of the biggest microcosms is the human gut (more than 10^13^ bacteria reside in the colon); gut microbiota interactions occur directly through binding by receptors to microbial ligands or indirectly by other factors that are produced by gut microbiota. This results in alteration of immune response, susceptibility to or protection against inflammatory diseases (Round and Mazmanian, 2009; Sommer and Backhed, 2013). For example, a seminal study by Rakoff-Nahoum et al. (2004) showed that the presence of commensal bacteria produce certain ligands like lipopolysaccharides (LPS) and lipoteichoic acid (LTA) that are sensed by toll-like receptors (TLRs) in the gut epithelium, and protect intestinal epithelium against injury. Also, microbial products released by certain microbes in the gut alter interactions between other microbes and gut. For example, colonization of germ-free mice by commensal bacteria was found to induce production of a particular C-type lectin, REG3γ, which has antimicrobial activity (Cash et al., 2006), particularly against gram-positive bacteria and thus indirectly affects interactions between other microbes and the gut. On the other hand, many commensal gut-associated strains also directly affect the gut by triggering a key nuclear receptor, PPARγ, which plays a major role in metabolism and inflammation within the gut (Nepelska et al., 2017). In addition, a commensal bacterium, *Fusobacterium nucleatum*, had the indirect effect of promoting human colorectal cancer cell proliferation and thereby leading to colorectal cancer (Rubinstein et al., 2013). These direct and indirect interactions are critical for modulation of the immune status of the host and susceptibility to disease. Moreover, the vagus nerve, the principal component of the autonomic nervous system modulates gut microbiota by slowing the cholinergic anti-inflammatory pathway which decreases intestinal permeability and shapes gut microbes. But under stress, this pathway is inhibited and increases the risk of the pathophysiology of irritable bowel syndrome and inflammatory bowel disease (Bonaz et al., 2018; Figure 2). In many cases, the configuration of the microbiome can significantly affect host-microbe interactions, leading to positive or negative physiological effects within the host and through our nervous system.

Diet plays a significant role in shaping the composition of gut microbes both on a short- and a long-term scale. For example, the short-term consumption of an animal-based diet rapidly increased the abundance of bile-tolerant *Bacteroides*, *Alistipes* and *Bilophila* and reduced abundance of carbohydrate-metabolizing Firmicutes such as *Roseburia*, *Eubacterium rectale* and *Ruminococcus bromii* over 1–2 days in humans (David et al., 2014). Moreover, rapid alterations in the composition of the gut microbiota were observed within 2–3 days with different sources of indigestible carbohydrate or fiber, diversity increased with diet rich in fiber from wheat bran (Walker et al., 2010).

Despite such dynamic shifts in the gut microbiome, habitual dietary patterns and inter-individual variations, such as genetic variations, appear to be primary determinants of the microbial composition. Long-term dietary patterns consisting of high-fat/animal protein and high-carbohydrate consumption have been broadly associated with microbial enterotypes enriched in *Bacteroides* and *Prevotella*, respectively (Arumugam et al., 2011; Wu et al., 2011). Although these responses were observed within 24 h, enterotype clusters did not switch in some individuals after 10 days of feeding (Wu et al., 2011). In addition, the gut microbiota diversity was increased after high-fiber, low-calorie diet only in individuals with reduced gene content of the gut microbiome compared to those with elevated gene content (Cotillard et al., 2013). Diet-host interactions have also been demonstrated with trimethylamine-*N*-oxide (TMAO), a hepatic oxidation product of the gut microbiome-generated trimethylamine from consumption of choline and carnitine found in eggs and beef. Considerable inter-individual variations in circulating and urinary TMAO concentrations have been reported, with high-TMAO producers following egg and beef consumption characterized by lower microbial diversity and greater enrichment of *Firmicutes* relative to *Bacteroidetes* (Cho et al., 2017). Our gut, food, and microbiome are also connected with our nervous system. Humans might get their prenatal microbiome (Aagaard et al., 2014; Parnell et al., 2017; D’Argenio, 2018) or at least be exposed to some bacteria (de Goffau et al., 2019) while in the uterus, and it is suggested that the gut-brain-bidirectional axis originates *in utero* (Borre et al., 2014; Jasarevic et al., 2017; Martin et al., 2018). Our gut and brain communicate via neurobiochemical, neuroendocrinal, and neuroimmune mechanisms, which are still unclear, and can be the result of different stages of development. Recent studies suggest that neurobehavioral outcomes can be influenced by: cytokine imbalance, vagal nerve signaling and hypothalamic-pituitary-adrenal (HPA) axis (Liu and Zhu, 2018; Martin et al., 2018; Figure 2). A growing list of neurobiological disorders includes autism spectrum disorders, schizophrenia, Parkinson’s disease, multiple sclerosis, bipolar mood disorders, anxiety, and depression have been associated with the gut-brain axis (Liu and Zhu, 2018; Martin et al., 2018).

## Evolution and Ecology of the Microbiome

### Evolution of the Microbiome

A host and its associated microbiota have profound effects on each other’s fitness, resulting in co-evolutionary processes that are still not well understood. The microbiome can evolve at two levels: first, each individual microbe is subject to evolutionary processes (mutation, selection, migration, drift, speciation, etc.), and second, a host species’ microbiome can evolve by incorporation and elimination of microbial taxa, or by changes in their relative abundances as a consequence of these evolutionary processes.

The microbiome evolved slowly and in a clock-like manner in the different branches of the great ape phylogeny, with the exception of a rapid depletion of diversity in the human lineage, which is thought to be associated with the consumption of meat (Moeller et al., 2014). Interestingly, mammals that have independently evolved an herbivorous diet often exhibit similar microbiomes (Ley et al., 2008; Muegge et al., 2011); however, this is not the case of panda bears, whose microbiome resembles that of their carnivorous and omnivorous close relatives, despite the panda’s herbivorous diet, probably due to phylogenetic constraints (Ley et al., 2008).

Within most mammals, the compositional overlap between the gut microbiotas of species populations in the Western hemisphere correlates with their geographic proximity, and each geographic location exhibits a characteristic microbiome composition that is not attributable to the diets or the evolutionary histories of the mammals living therein, suggesting that horizontal transmission also shapes the microbiome (Moeller et al., 2017). This relationship is most evident in sympatric predator-prey populations due to one species and their associated microbiota serving as the diet for the paired predator. The structure of the associations is unclear in primate species but will likely display some similar trends.

### Intra-Species Microbial Diversity

The composition of the microbiota within a species can vary significantly due to complex behaviors of the host. Environmental pressures derived from host-associated behaviors such as diet and exposure to medicine and antimicrobial compounds heavily influence the prevalence of microbial species within a host-associated community (De Filippo et al., 2010). Major nutritional shifts between a traditional diet, which is high in fiber, and an industrialized ‘Western’ diet that is high in oils, refined sugar, fatty meats, and salt correlate with the prevalence of certain microbial taxa. For example, *Bacteroides* and *Firmicutes* dominate the gut microbiome of healthy people on industrialized diet (The Human Microbiome Project Consortium, 2012; Yatsunenko et al., 2012; Lloyd-Price et al., 2016), whereas species commonly attributed to disease states such as *Prevotella* and the spirochete *Treponema* (Wu et al., 2011; Schnorr et al., 2014; Obregon-Tito et al., 2015) are more common in people relying on traditional diets. Importantly, switching between these diets during societal industrialization can lead to detectable changes in the microbiome but require long-term dietary shifts to be maintained (Wu et al., 2011; Gomez et al., 2016). Ingestion of antibiotics, in contrast, has immediate and severe consequences, decreasing the taxonomic diversity, richness, and evenness by up to 30% (Dethlefsen et al., 2008; Dethlefsen and Relman, 2011). Recovery of the initial microbial diversity following an antimicrobial selective sweep may occur quickly in some individuals whereas others experience dysbiosis, i.e., disturbances in composition and function, for months or years (Buffie et al., 2012; Fonseca et al., 2015; Wipperman et al., 2017). Thus, initial insults to the microbiota that disrupt the stable selective pressures maintaining a homeostatic balance lead to major changes in the distribution of taxa in the gut with serious consequences for the host.

In addition to environmental pressures on evolution of the microbiome, intrinsic genetic mechanisms likely play a key role in shaping microbial diversity. Yet, substantial barriers exist to accurately measuring intra-species diversity within microbial communities and, consequently, they have been largely ignored. Numerous *in vitro* evolution and environmental microbial community studies have demonstrated the dynamics of new genetic variants emerging and quickly sweeping across complex populations (Denef and Banfield, 2012; Jerison and Desai, 2015; Levy et al., 2015; Bendall et al., 2016). Interestingly, these population dynamics mirror the same selective sweeps that arise following perturbation by antibiotic compounds in the gut. We posit that variation observed within single individual hosts over time (Caporaso et al., 2011; Gajer et al., 2012) may reflect not only alterations in the microbiota due to changes in diet or other external perturbations but also competition within the host niche that produces shifts in the relative proportion of different taxa, consistent with the “ecosystem on a leash” model (Foster et al., 2017). As mutations arise in resident microbes, their relative fitness may increase or decrease leading to alterations in the composition of the microbiome. In *Candida albicans*, a common fungal commensal, strains harboring a single nonsense mutation in *EFG1* have an advantage over strain with an intact *EFG1* and quickly outcompete wild type strains in the GI (Pierce and Kumamoto, 2012; Pande et al., 2013; Hirakawa et al., 2015). Preliminary studies suggest that these evolutionary dynamics do occur within the gut microbiome and we are beginning to construct methodologies to directly measure mutation rates (Garud et al., 2017).

Single species within the GI can exert selective pressure on the composition of the rest of the microbiome. Distinct GI communities of “enterotypes” are centered on key bacterial species such as *Bacteroides* (enterotype 1) and *Prevotella* (enterotype 2) although each enterotype spans a range of species prevalence (Arumugam et al., 2011; Gorvitovskaia et al., 2016). Additional microbes delineate these microbiome signatures that likely reflect species-species interactions (Boon et al., 2014; Zelezniak et al., 2015). These interactions are often difficult to define in complex gut communities but have been identified through *in vitro* and germ-free animal approaches for toxin secretion (Chatzidaki-Livanis et al., 2014; Hecht et al., 2016), shared metabolite cycling (Fischbach and Sonnenburg, 2011; Zelezniak et al., 2015), and niche specialization (Mahowald et al., 2009; Hibbing et al., 2010). The presence of other nearby microbes within the GI can alter the transcriptional profile of different species, suggesting microbial crosstalk that regulates some of these interactions (Plichta et al., 2016). Thus, selective pressure through inherited and contemporary interactions through life likely plays a prominent role in establishment of a microbiome and resilience to disruptive events.

More recently, an alternative view of species abundance and competition has emerged that focuses more on the genes encoded within the genomes of resident microbes and less on taxonomical units. Microbes within communities tend to have reduced genome size, relying on the surrounding microbes to provide some of the metabolites required for growth as described in the Black Queen Hypothesis (Ochman and Davalos, 2006; Morris et al., 2012; Boon et al., 2014). The presence of genes encoding different clusters of enzymes are central to this view of the microbiome as an interdependent metabolic network that can distinguish individual variation (Bradley and Pollard, 2017). Complications linking the resident microbes to these enzymes arise from the spread of genes by lateral gene transfer, LGT or also known as horizontal gene transfer, HGT within the tightly associated gut microbiome (Smillie et al., 2011). Importantly, high rates of LGT within the human microbiome pose clear health risks to resemble a reservoir for antimicrobial resistance (AMR) genes (Sommer et al., 2009, 2010). The accumulation of these genes likely stems from widespread and often unnecessary use of antibiotics in the industrialized world that selects for LGT of AMR cassettes.

## Methods of Studying the Microbiome

Microbiome research is a highly transdisciplinary field with a wide range of applications and methods for studying it. In this section, we identify several important methods (computational approaches and models) for obtaining microbiome data, discuss several widely used mathematical and computational techniques to analyze microbiome data, and further understand the functions and role of the microbiome.

### Disparate Models and Versatile Methods

The current arena of microbiome research shows disparately diverse models anchored to the customs of each specific discipline in terms of modeling efforts. Ecological, epidemiological, and physical sciences have tackled the problem of understanding the microbiome at very different spatial and temporal scales (say from genes to populations, and from nanosecond to seasonal variability) and trying to find a general, perhaps unifying, modeling paradigm in microbiome research initially appears daunting and unproductive. Here, we focus on methods that, in our humble opinion, are tackling different objectives and modeling philosophies. However, the methods that are currently employed to analyze or predictive microbiome features at different spatio-temporal scales can likely be applied at other scales or integrated among each other; and this constitutes itself a computational and biological avenue for research.

### Properties of Microbiome Data and Considerations Regarding Collection Strategy

The microbiome is commonly studied through a variety of high-throughput cultivation independent techniques. These include using next generation sequencing to identify the genetic material of the microbes, and additional ‘omic technologies to identify the functional products of the microbes, such as metaproteomics for proteins (Hettich et al., 2013), metatranscriptomics for gene expression (Bashiardes et al., 2016), and metabolomics for small molecules (Nicholson et al., 2005). All of these techniques probe a different aspect of the microbiome and generate large amounts of data that is processed and analyzed to infer information about the microbial communities. Sophisticated bioinformatics and mathematical methods are needed to extract meaningful information and conclusions from these data.

Most commonly, the microbiome is studied using sequencing by one of two approaches: metagenome sequencing and marker gene sequencing (Weinstock, 2012). Metagenome sequencing aims to sequence all of the microbial genes in a given sample and provides insights into the composition and genetic repertoire of the microbiota, while marker gene sequencing aims to sequence a specific gene region, such as the 16S ribosomal RNA (rRNA) gene that is specific to bacteria and archaea, and it gives a broad picture of the types of microbes present. While both strategies give information about the microbial composition of the microbiome, there are distinct differences and benefits for each approach. Sequencing entire DNA from a sample–microbial or not–requires greater sequencing depth per sample. This leads to a more complete picture of the genetic contents of the microbiome and allows researchers to assess the functional potential of the microbiome based on gene function and begins to address population dynamics during longitudinal sampling. However, metagenome sequencing generates large volumes of data that requires more computationally intensive analysis than marker gene sequencing. Since marker gene sequencing is restricted to a specific site of the genome, much less sequencing depth is required to characterize the microbial communities in a sample, and hundreds of samples can be combined onto a single sequencing run (Caporaso et al., 2012). This reduces the computational and overall cost dramatically, but only allows for the relative abundances of targeted organisms to be identified at reduced taxonomic resolution. Selection of an optimal amplification primer, however, is crucial to both limiting the introduction of bias in relative abundances, which may be caused by a primer’s lack of sensitivity to certain organisms (Bergmann et al., 2011; Hayashi et al., 2013), and maximizing coverage over a microbial community (Bergmann et al., 2011).

Although microbial sequencing surveys will continue to advance the field, microbiome research is beginning to focus on the function and mechanistic aspects of microbial communities (Waldor et al., 2015). Metabolomics is one of the key technologies that promises to assist in the understanding the function of the microbiome (Dorrestein et al., 2014; Gilbert et al., 2016; Knight et al., 2018). There are two basic approaches to metabolomics (Gilbert et al., 2016; Melnik et al., 2017). First is the targeted metabolomics, where analysis is performed with a pre-determined list of molecules (Melnik et al., 2017). The strength of this strategy is that one can target specifically and therefore it is more sensitive and quantification is of higher quality. The most common mass spectrometry instrument to use for this are triple quadrupoles. When samples become very complex, however, such as fecal samples that contain environmental, xenobiotics such as drugs and personal care products, food, food packaging, microbial, host and various metabolized versions of molecules, one needs to be careful not to over interpret the data even when co-migration with an authentic standard is performed (Melnik et al., 2017). Further, with a pre-determined list of candidate molecules that are investigated, it will not be possible to discover molecules that are not in the pre-determined list or discover specific molecules not yet described. Generally, a targeted metabolomics experiment aims to find molecules that belong to well-described pathways and with standards that are commercially available. The second strategy is untargeted mass spectrometry. In untargeted metabolomics there is no list of defined molecules that are investigated, but rather it is a strategy that lets the data inform on the molecules that are detected. Once the data is collected it can be used in a broad sense to show how the overall molecular profiles are changing or attempts are made to annotate the observed molecules. Annotations are accomplished by matching the data to reference libraries and to *in silico* predictions. Because there is a very small number of reference spectra in the public domain compared to number of known structures, the annotation rates are still low. For well-studied samples such as human plasma the annotation rate may be as high as 10% while the annotation rate for fecal samples is halved while for soil or ocean samples the annotation rates are below 1%. Also, one still has to manually inspect the annotations and the most important annotations will need to be confirmed with standards. However, manual inspection is not scalable and there is an inherent bias in the reference libraries toward commercially available molecules.

To address the scalability, the first algorithms that enable false discovery estimations are being developed (Jiang et al., 2017; Waldron, 2018). Because most microbially derived molecules, especially natural products, are not commercially available, there has been a dearth of widely available annotation data for such molecules, limiting the detectability of such molecules during annotation-based analyses, and leading to an inherent bias against understanding such molecules’ functions. To overcome this limitation a global natural products social molecular networking infrastructure was created, allowing users to annotate mass spectra directly in their data. When an annotation is made it becomes a part of the public Global Natural Products Social Molecular Networking (GNPS) contributed reference collection (Wang et al., 2016). This is improving the amount of public knowledge exists for the annotation of microbiome derived molecules. Such annotations may also be propagated using a molecular networking strategy. Another strategy is through *in silico* predictions. Dereplication against a database, including CSI:FingerID (Dührkop et al., 2015), Metfrag (Wolf et al., 2010), Metfusion (Gerlich and Neumann, 2013), CFM-ID (Allen et al., 2014), Network Annotation Propagation (da Silva et al., 2018), dereplicator (Mohimani et al., 2016), and dereplicator + (Mohimani et al., 2018), aims to accomplish *in silico* prediction, with dereplicator + being the only approach specifically tested against microbial data to date (Mohimani et al., 2018). Interestingly much of the data that is observed in an untargeted mass spectrometry experiment, including natural products, do not fall into the traditional biochemical pathway maps (KEGG is one such map) while often the molecules targeted do fit such pathways, highlighting the complementarity of the strategies.

### Statistical Analysis, the Microbiome, and the Importance of Data Normalization

Marker gene surveys of the microbiome are frequently used to broadly understand microbial communities. In these studies, the samples, which are processed through sequencing and bioinformatics pipeline, are summarized as a table of operational taxonomic unit (OTU) counts. Statistical analysis typically starts with this OTU table, which is sparse, high dimensional and exhibits dramatic variability in the total number of counts (called library size) across samples. These microbiome-specific data properties have serious implications on data analyses, where popular first line approaches, such as Principal Coordinates Analysis (PCoA), are not designed to deal with such extreme levels of sparsity and heterogeneity (Warton et al., 2012; McMurdie and Holmes, 2014). Two particular implications include much larger number of principal components (up to 60 in some data sets) required to explain a reasonable amount (at least 70%) of data variability and misleading estimates of sample-wise dissimilarities, or beta diversity. These challenges create immediate problems with data visualization since: (1) two- or three-dimensional data reduction plots often express only a small proportion of variability that may limit scientific conclusions from limited resolution; and (2) the data should be properly normalized, before downstream analysis, to evaluate differences between groups of samples with different conditions (e.g., healthy versus control). Thus, extreme care should be taken in data processing before analysis. Counts tables should be properly normalized, uninformative and potential contaminant OTU features filtered out, and proper statistical methods designed specifically for microbiome analysis, such as methods corrected for over-dispersion, should be used (Alekseyenko, 2016). One way to deal with the sparse count data and the large number of OTUs is to incorporate the phylogenetic tree structure in defining the distance between two microbial communities, which provide a biologically interpretable method of pooling rare OTUs. Important examples of such distances include weighted and unweighted UniFrac distances and its generalizations. These phylogenetic tree-based distance matrices can be used in PCoA.

Therefore, to assign 16S rRNA sequencing reads to a set of *p* known taxa, for example at genus level for each sample, the raw data must be summarized as an *n* x *p* dimensional matrix of read counts, denoted by *X*, where *n* is the number of samples. Such a matrix is often sparse with many zero elements. Such zeros can be due to dropouts during sample preparation steps or due to under-sampling due to limited sequencing depths. The number of zeros observed in a given sample is often inversely associated with the sequencing depths, suggesting many zeros are due to under-sampling (Cao et al., 2017). In addition, the read counts vary greatly from taxon to taxon, with some very large read counts often being observed for a few taxa.

In order to make resulting taxon counts comparable across different samples, the count matrix *X* is often converted into a matrix of proportions by dividing each row by the row total. If this normalization essentially assumes that for each sample, the counts data follow a multinomial distribution conditioning on the total number of reads observed. The empirical proportions are the maximum likelihood estimates of the multinomial probabilities. Such a simple normalization has two drawbacks. First, the typical multinomial distribution usually does not fit the observed read count data well in microbiome studies due to both excessive zeros and also some very large counts in a few taxa. Alternatively, Dirichlet multinomial or zero-inflated Dirichlet multinomial distributions can be assumed for the observed data and can be used for normalizing the counts across samples (Holmes et al., 2012). Second, such parametric models cannot differentiate structural zeros from zeros due to under-sampling. In addition, the models might not be flexible enough to capture the dependency structure of the true compositional matrix. There have been some attempts of using the methods developed for RNA-seq data such as edgeR or DEseq to normalize the microbiome count data (Weiss et al., 2017). However, neither of the models fits the microbiome data due to excessive zeros observed. These parametric models are mainly developed for directly modeling the count data for differential abundance analysis, rather than for normalizing the count data into proportions. Among these parametric methods, zero-inflated Dirichlet multinomial distributions provide the most flexibility and fit the microbiome counts better than other models (Tang and Chen, 2019).

Microbiome count data have some similarity to the count data observed in single cell RNA sequencing (scRNA-seq) data in term of excessive zeros due to dropouts. However, many existing scRNA-seq normalization methods assume existence of spike-in data, which are not available in microbiome studies (Vallejos et al., 2017). If the truth is that different samples indeed have different numbers of bacterial taxa, it is difficult to develop a normalization method to account for such differences. This is again different from RNA-seq data since the number of expressed genes is often the same across different samples. If one assumes that the observed zeros are indeed due to dropouts or under-sampling and all the samples includes the same set of taxa, allowing some bacterial taxa at very low abundances, it is possible to take a multi-sample approach to obtain better estimation of the relative abundance matrix and to make the data across samples comparable. Cao et al. (2017) presented an effective empirical Bayes method that borrows information across samples to obtain more accurate estimate of the compositional matrix. Due to large noise in the data, it is a good practice to eliminate the taxa with very small counts before performing data normalization.

While many of the most-salient normalization issues for 16S rRNA sequencing data have been discussed above, there is also a great need for better normalization methods for metagenome sequencing data. For such data, normalization can be applied at several levels, including taxonomy, gene level and the pathway level. At the species level, one can align the reads to a set of universal marker genes or a set of clade-specific marker genes (Segata et al., 2012). Like 16S rRNA read counts, how to handle excessive zero in the data is not clear. For microbial genes or gene sets, since there are not many zeros in the counts, standard normalization methods such as reads per kilobase million (RPKM) can be applied.

### Pattern-Oriented Models

Pattern-oriented models are typically assumption-free models that are concentrated on finding the necessary and sufficient information to reproduce the observed or designed patterns in complex ecosystems (Coyte et al., 2015; Zeng et al., 2015; Servadio and Convertino, 2018). Patterns are meaningful, stable, and potentially universal probabilistic relationships reflecting the collective dynamics of complex ecosystems, such as biodiversity patterns dependent on microbiome- and microbiome-environment interactions. These patterns are the foundation of metacommunity modeling. This theoretical and computational modeling philosophy, applicable also in microbiome research for predicting macroecological patterns (Li and Convertino, 2019), is in striking contrast with statistical and mechanistic process-oriented models that are anchored to traditional probability theory or reductionist modeling approaches aiming to mimic precisely the assumed mechanisms, and to preserve the full set of data (thought as uncertainty free) as they are. These mechanistic models typically rely on completely hypothesized processes about the functioning of complex ecosystems such as the microbiome (Hubbell, 2006); mechanisms that are hard to verify if prediction accuracy is the only endpoint to consider. Here we focus our attention on information and network theoretic models (Servadio and Convertino, 2018; Li and Convertino, 2019) that draw their foundational concepts and analytics on statistical physics (or complexity science at large) and define the most relevant input data to characterize macroecological patterns (Li and Convertino, 2019). Information theory is mostly useful when predicting microbiome patterns by simplifying the complexity of the problems and linking driving factors to outcomes considering their whole probability distributions, which can explore the complexity of the microbiome. In particular we are interested in pattern-oriented models that aim to extract the ecological and environmental information of microbial communities from data and implement that into simple macroecological models for predictive purposes [see also Convertino et al. (2009) and Zeng et al. (2015) as well as Li and Convertino (2019) for characterizing the linkage between species interaction networks and macroecological patterns].

Specifically, these models aim to infer the optimal functional and structural networks of microbes related to patterns of interest for the studied animal and human populations (Li and Convertino, 2019), as well as multiplex networks among multiple populations or between the microbiome and the environment. Other goals of these models include the identification of local environmental features driving the ecology and evolution of microbiome networks considering the observed patterns (of microbiome composition or other associated patterns such as of diseases) (Parfrey and Knight, 2012; Martí et al., 2017), potential feasible patterns and state transitions which may be associated to environmental exposures (Bucci et al., 2012; Bashan et al., 2016), and symptoms or diseases (Martí et al., 2017). These functional and/or structural networks can be the basis of verified co-occurrence networks as defined later (see section “Harnessing Association Graphs to Discover Co-occurrence Relationships”).

**FIGURE 3:**
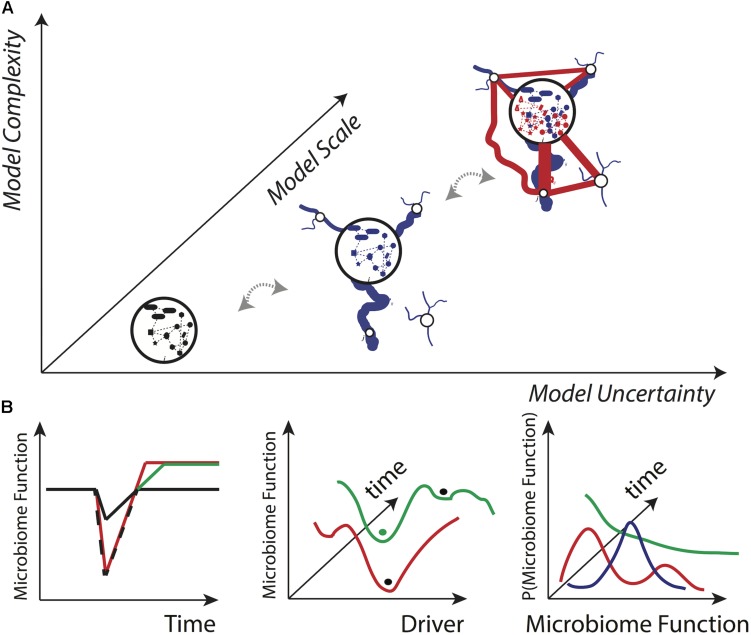
Conceptual model complexity-uncertainty-scale manifold and desirable model outputs. **(A)** According to general computational complexity principles, it is expected that microbiome uncertainty (information) grows with the spatio-temporal scale of analysis and the complexity of the system (data) analyzed. These principles are independent of model and microbial systems. The scale is the biological, spatial and/or temporal level of analysis and defines the sensitivity (variability) of the model. For instance, at micro-, meso-, and macro-scales the analysis can be at the individual, population and multi-population level. The scale also defines information complexity that may be related to potentially causal networks for the microbiome such as natural and human spreading networks (in blue and red). Each node of the community details a microbiome interaction network or graph. **(B)** Three outputs of general interest in microbiome research for assessing systemic risk and resilience that have an increasing focus on systemic properties, from left to right: microbiome feature value over time (e.g., function), microbiome feature state-space over a gradient of drivers, and systemic probability distribution of microbiome features under different scenarios.

Figure 3A shows the typical modeling trade-off between model complexity, uncertainty and scale (see Convertino and Valverde, 2019), as well as potential plots of interest for microbiome research generated by information theoretic or other stochastic models. These plots, in order of information significance from left to right, refer to key features of the microbiome, such as microbiome functional diversity, which is known to affect health and disease in populations (Martí et al., 2017; Li and Convertino, 2019). In Figure 3B, the first plot on the left shows a typical ‘’stress response” profile where the stress-dependent response of the microbiome is evaluated at a singular time point; the persistence of these fluctuations typically shows the resilience of the microbiome against one or multiple external stressors (Shade et al., 2012) but cannot say anything about how the microbiome responds to different levels of the stressor. The middle plot of Figure 3B shows the microbiome variability over the gradient of a stressor or driver more generally (endogenous or exogenous); this plot has the utility to evidence the landscape of all potential states in relation to all drivers and more importantly to manageable factors, such as antibiotics (see, e.g., Bucci et al., 2012). The ‘’balls” in the plot identify a microbiome state that is related to a metastable, stable or unstable condition of the population considered (e.g., healthy or diseased). This second plot, however, cannot show how the microbiome is changing with respect to a stressor probabilistically speaking. Finally, the last plot in Figure 3B shows the whole probability distribution function (pdf) of a microbiome in relation to stochastic changes in the stressors; this plot is the most informative since any pdf can correspond to a ‘’microbiome state” as shown by the middle plot, and the whole range of values (with the corresponding probability) is identified. Typically, a ‘’state” is associated to a microbiome functional network that is reflected by a certain pdf type (Convertino and Valverde, 2019).

### Harnessing Association Graphs to Discover Co-occurrence Relationships

**FIGURE 4:**
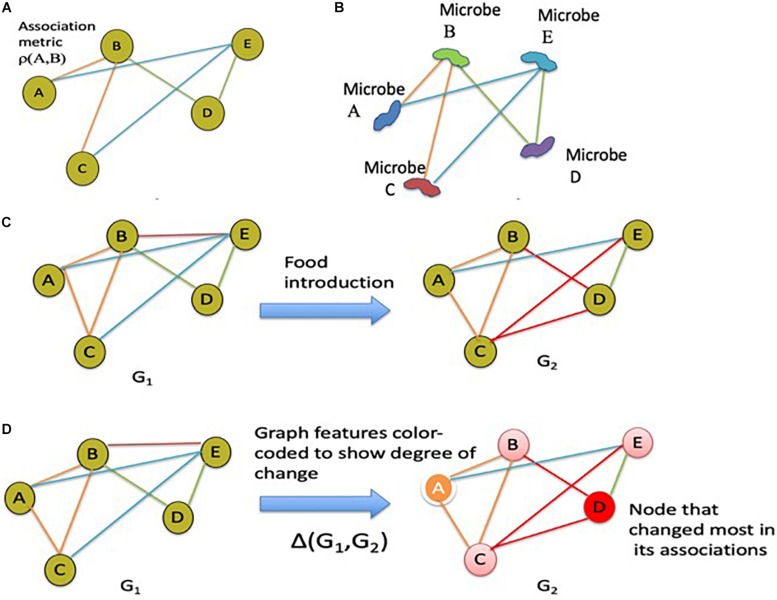
Association graphs demonstrating microbial co-occurrence networks and microbial composition changes over time. These association graphs can be included into metacommunity models such as the one in Figure 1. **(A)** Schematic diagram of an association graph. **(B)** Schematic microbial co-occurrence network based on the association graph shown in panel **A**. **(C)** Schematic showing the changes in gut microbiota associations within an individual in response to the introduction of food; other external stressors can be considered equivalently. **(D)** Variation of the schematic shown in panel **C** with color-coding to show the degree of change at each node.

Association graphs (or networks as named before) are widely used in informatics (Zunde, 1971; Pearl and Wermuth, 1994; Pelillo et al., 1998; Bartoli et al., 2000; Jayadevaprakash et al., 2005; Balaneshinkordan and Kotov, 2016; Liu and Shen, 2016; Mehler et al., 2016; Luo et al., 2017) to discover and learn interrelationships between key agents that make up a complex system. Historically, association graphs have been used in text-based information retrieval (Jayadevaprakash et al., 2005; Balaneshinkordan and Kotov, 2016; Mehler et al., 2016), hierarchical pattern analysis (Pelillo et al., 1998), interpretation of data models (Zunde, 1971; Pearl and Wermuth, 1994), and recently, in a diverse range of multi-variate data informatics applications (Hosseinkhani et al., 2012; Date et al., 2013; Liu and Shen, 2016; Luo et al., 2017). For example, such associations have been employed for context mining in crime diagnostics applications (Hosseinkhani et al., 2012). More recently, association analysis and other computational techniques to determine interrelationships between pathogens and their ecosystem have been proposed in the context of microbial networks (Jiang and Hu, 2016; Baker et al., 2018; Park et al., 2018). Computational methods that employ association graphs to discover co-occurrence relationships between pathogens in the environment (Jiang and Hu, 2016; Baker et al., 2018; Park et al., 2018), could be employed in the context of the gut microbiome. For example, association graphs could be used to discover and track co-occurrence associations between gut microbes of infants being introduced to solid foods. Specifically, a multi-scale architecture for developing association graphs across both longitudinal and aggregate studies may be constructed. Figures 4A,B provide the schematic idea for microbial co-occurrence networks for the infant gut microbiome based on association graphs. Figures 4C,D provide the schematic diagram showing how the changes across the gut microbiome of a single infant can be tracked over different times and event milestones (e.g., introduction of a particular solid food) using distances between the association graphs. Data can be extended to multi-scale graph architecture connecting the association graphs tracked across individual longitudinal studies to aggregate studies across larger cohort of subjects, to tease out microbial agents that change over time and other factors. Association graphs by themselves, however, do not provide a computational means for inference modeling or an ecological view of microbial interactions. Network inference models may be employed to achieve these aims.

### Network Inference Models

There are numerous network inference models that could provide value in the study of the microbiome. Information theoretic models, for example, can be used for inferring microbial interaction networks (Li and Convertino, 2018); these models are not based on any particular assumption about microbial dynamics and are simply based on the probabilistic characterization of species abundance and their evolution over time and in space (where the spatial domain is involved). Additionally, because of the entropy-based nature of these models, relevant information is also extracted from the original microbial data (Servadio and Convertino, 2018; Li and Convertino, 2019) in order to infer the most stable network considering the complexity-uncertainty-sensitivity information landscape. In these models, the inferred interactions are not necessarily revealing truly causal feedbacks between microbes, but the models’ output can often be used for biological research and microbiome engineering. The inference of interactions is based on transfer entropy (TE) (Schreiber, 2000), which is an information theoretic and non-parametric function referring to the directed exchange of information between two variables (species abundance). This function describes the directional communication from a source to a destination, with species in the microbiome network each represented as a node.

## Diagnostics and Interventions Using the Microbiome

Understanding the microbiome can enable us to use it as an evaluative tool. If we can thoroughly understand the relationship between the state of the microbiome and biological processes (e.g., disease, wound repair, organ function) within the host, we can look to the microbiome as a robust, low-cost diagnostic tool to quickly identify dysfunction in such biological processes and to remediate problems earlier than we otherwise could. Moreover, the microbiome could potentially serve as a classification tool using sample composition to gain insight into samples’ origins and history. In this section, we discuss several potential applications of the microbiome for diagnostics and classification.

### Gut Microbiome as Diagnostic and Prognostic Tools

Research in the last decade has focused on the use of the microbiome as a potential disease classification, diagnosis and prognosis tool. Among these, the gut microbiome has been mostly studied. Examples of using gut microbiome as a possible diagnosis tool include inflammatory bowel disease (Gevers et al., 2014; Zhou et al., 2018), progression of diabetes (Leustean et al., 2018), and irritable bowel syndrome (Hollister et al., 2019). Statistical methods to build such prognostic tools include random forests and penalized regression analysis that can take into account the compositional nature of the microbiome data (Shi et al., 2016; Lu et al., 2019). Alternatively, for shotgun metagenomic data, a combination of reference-based known microbial abundance characterization together with assembly binning-based known organism feature extraction has been shown to improve the prediction of several diseases such as colon cancer and type 2 diabetes (Zhu et al., 2019).

### Dermatological Diagnostics and Tools

While many diagnostics have been made for the gut, the skin is the most accessible human organ, and therefore, a natural target for diagnostic sampling.

#### Skin Microbiome

The skin is the human body’s largest organ, and it is fully exposed to the external environment. Consequently, the skin is the first barrier of the human body and the first host of microbiomes coming from the external environment. Its surface is therefore inhabited by a plethora of microbial agents that vary in genetic makeup and function in relation to the skin. Some of these resident microorganisms are merely bystanders while others work together with skin in a mutualistic relationship such as to boost the immune system. This skin flora, or microbiome, is crucial for healthy skin; yet harmful, pathogenic agents sometimes invade the flora and cause damage, disease, and slow healing. Understanding this microbiome, is the key to understanding how to protect and maintain healthy skin. Here, we discuss current methods of obtaining and analyzing skin microbiome samples and predict a future direction for diagnostic technology in this field.

#### Diagnostic Methods for the Skin

Swabbing has been previously shown to be just as effective at collecting representative bacterial flora as more invasive methods like scraping and punching biopsies (Grice et al., 2008). In fact, using swabbing over more invasive techniques leads to better sampling of the micro-organism DNA rather than the host DNA, which would be highly prevalent in a skin biopsy. Swabbing involves a sterile cotton swab immersed in saline and polysorbate buffer to stroke across a selected area of skin for a number of times. The swab is then placed directly into the storage buffer and held at −20°C until ready for extracting DNA or further processing. One of the challenges with trying to obtain DNA from skin for bacterial identification over the typical specimen of stool is that the bioburden is much lower and thus the materials used for the DNA detection must be optimized.

Processing of samples include cell counting and extraction of DNA/RNA. To identify target cells, fluorescence *in situ* hybridization (FISH) with probes for the 16S rRNA gene sequence can be used to visualize bacteria (Fortner et al., 2014). Stains like propidium iodide and thiazole orange may be used to differentiate between viable and non-viable cells. The bacteria may then be counted on a hemocytometer or by flow cytometry.

Bacteria in the samples may also have their DNA extracted for genetic analysis. Generally, standard DNA extraction protocols (lysis in extraction buffer) are followed with polymerase chain reaction (PCR) amplification of the 16S rRNA gene (Grice et al., 2010). Real-time PCR (also known as quantitative PCR or qPCR) may also be used to quantify the target. The DNA may then be sent for sequencing and phylogenic analysis of the 16S rRNA gene to differentiate the strains of bacteria (bacterial species have been defined as having ≥97% identical 16S rRNA gene sequence) (Konstantinidis et al., 2006). Microarrays may also be used if probes for bacteria are known.

In addition to DNA extraction and analysis, immunoassays may be used to probe specific strains of bacteria directly with high sensitivity (can also be made portable but requires a stable antibody). Recent development of cell-binding antimicrobial peptides (AMP) may function similarly to antibodies in immunoassays, yet maintain greater robustness and broader specificity, though more validation of these AMPs may be needed (Mannoor et al., 2010).

As an alternative to skin swabbing, adhesive tapes can be used to non-invasively collect samples of superficial layers of the epidermal stratum corneum and residing bacteria. Such adhesive tapes generally consist of an adhesive agent bound to a tape backing which may be applied on the skin and subsequently peeled. Recent commercial implementations have been shown to be as effective as swabbing for collecting microbiome (Yao et al., 2017). Moreover, it has been demonstrated that an adhesive may be applied on the skin in a way that does not change the skin’s cytokine profile or cause inflammatory cell migration from vasculature to the dermis/epidermis during the first few hours following application of the adhesive (Rheins and Morhenn, 2005; Benson, 2011), which otherwise could alter the microbial composition of the collected sample from its natural state. Consequently, use of an adhesive for collecting samples may help streamline the process of sample collection by standardizing the collection process and simplifying storage and transfer.

#### Future Directions

In the future, one could anticipate treatment devices such as bandages and wound dressings that also function as diagnostic in nature, such as a bandage within which DNA from bacterial can be extracted and analyzed (Rheins and Morhenn, 2005; Benson, 2011). Future studies may include comparing the DNA extraction obtained from various sampling methods from swabs to adhesive patches to alternative devices. There might even be technologies by which these devices could analyze the materials in real-time. For instance, colorimetric assays imbedded in the bandages may be used to detect bacterial metabolomics. Because bacteria have distinct metabolic requirements, their metabolites may be used as a biomarker for indirectly detecting and characterizing the microbiome. Further studies will be needed to describe correlations between metabolites and microbiome and to validate the metabolic signatures, but future technology may incorporate analytical measures such as colorimetry of bio-signatures, either *ex situ* after samples have been collected or *in situ* and real-time via adhesive bandage to characterize the skin’s microbiome. A metabolomics approach may be advantageous over direct detection of bacteria as detection of biomarkers can be simpler and less restrictive with respect to analytical detection methodology and more generally applied to various bacterium.

Other methods to explore include use of radio frequency identification (RFID) signal fluctuations to detect in real-time the presence or absence of bacteria, though specific information on bacterial strains have not been demonstrated (ElMahgoub and Shaban, 2014). Additionally, an auto-fluorescent device utilizing a broadband white LED output and dual-wavelength LED detection array may be capable of real-time detection of bacteria and is currently in preclinical studies (Wu et al., 2014).

### Microbial Forensics

Physical and chemical features have traditionally been used for forensic analysis of samples (Katz and Halámek, 2016), however, these are not always sufficient to characterize samples (due to intentional and non-intentional contamination). Therefore, microbial signatures are being investigated due to their slower adaptation to contamination and their uniqueness within various environments (soil, human body sites, etc.), thus helping us to identify their origin (Hampton-Marcell et al., 2017). With the increasing possibility to sequence DNA “anywhere, anytime” using nanopore technology (Jain et al., 2016), microbial DNA can be used as a new type of “sensor” in environmental analyses. Microbes and DNA are ubiquitous and diverse, yet microbial communities exhibit repeatable patterns across many ecosystems and sample types (Relman, 2012). Furthermore, microbes exhibit different phenotypic and functional profiles that drive observed phenotypes, e.g., the biogeochemical processes in soil. Many of these features depend on geospatial environmental factors (e.g., climate), the presence or absence of other microorganisms, and availability of nutrients and space. Therefore, metagenomic sequencing (and metabolomics) can be useful forensic tools, even though these are still underdeveloped. Also, there is almost no information regarding how microbial signatures vary and/or are robust to chemical disturbances.

Current research has focused on geolocation, human microbial signature identification, and postmortem identification (Clarke et al., 2017). We know that environmental factors have large effects on microbial community structure (Bouchot et al., 2014). Therefore, climate, altitude, and land-use all can have a drastic effect on microbial community composition and function, as shown in sampling different cities (Afshinnekoo et al., 2015; O’Hara et al., 2017). Human microbial signatures are diverse across body parts (The Human Microbiome Project Consortium, 2012) and can be affected by a variety of lifestyle factors (Casarin et al., 2012; Yatsunenko et al., 2012; Bizzarro et al., 2013; Song et al., 2013; David et al., 2014; Kort et al., 2014; Moon et al., 2014; Misic et al., 2015). It has been shown that human skin microbial signatures dominate the indoor microbiome (Kembel et al., 2014; Chase et al., 2016), especially indoor air and HVACs (Meadow et al., 2015; Misic et al., 2015; Prussin and Marr, 2015). The gut microbiome can be collected from toilets, and individuals from diverse geographic locations can be differentiated both by specific microbial sequence signatures (Yooseph et al., 2015), and by 16S rRNA-based taxa composition (Yatsunenko et al., 2012). Moreover, postmortem intervals can be determined by the microbiomes’ turnover in the host’s decomposition (Damann et al., 2015), and in different body locations (Damann et al., 2015; Hauther et al., 2015; Johnson et al., 2016).

### Microbiome Interventional Strategies

In addition to the diagnostic methods described in the previous section, the microbiome may further be leveraged as an interventional tool. For example, in scenarios where a particular microbiome is well understood, we may be able to alter the microbiome to achieve a desired physiologic effect in the host or other effects within a larger environment. In this manner, we may use the microbiome as a lever to indirectly intervene in other processes within a microbiome-host system or even a larger-scale microbiome-environment system. Here, we discuss several examples where such an interventional strategy may be applied.

#### Prebiotics

As the gut microbiome holds great promise for modulating risk of chronic diseases (Shreiner et al., 2015), the International Scientific Association for Probiotics and Prebiotics (ISAPP) consensus panel has recently updated the definition of a prebiotic as a substrate that is selectively utilized by host microorganisms conferring a health benefit (Gibson et al., 2017). Non-digestible carbohydrates such as inulin, fructo-oligosaccharides (FOS) and galacto-oligosaccharides (GOS) are commonly used prebiotics; abundant in onions, asparagus, agave, artichoke, etc. They have been shown to increase the abundance of beneficial *Bifidobacterium* and/or *Lactobacillus* spp. (Gibson and Roberfroid, 1995; Thongaram et al., 2017), contain enzymes that aid in the digestion of FOS and GOS, and protect our gut from pathogens and relieve constipation (Mitmesser and Combs, 2017). Furthermore, the specificity of polysaccharide use among *Bacteroides* spp. has been linked to their proliferation in the presence of fructans (Sonnenburg et al., 2010), suggesting the response of the microbial community to dietary glycans. However, inconsistent effects are present across different studies using various durations, doses, dietary forms, and subject characteristics, and the efficacy of carbohydrate-based prebiotics is inconclusive (Gibson et al., 2017).

The updated ISAPP definition of prebiotics expands to include diverse substances such as non-carbohydrate-based products (Gibson et al., 2017) such as polyphenols and fatty acids, can also shift the microbial populations (Singh et al., 2017). Polyphenols are plant secondary metabolites that are known for their antioxidant properties (Scalbert et al., 2005), and many of these compounds have been associated with greater levels of *Bifidobacterium* and *Lactobacilli* and reduced levels of *Clostridium* spp. (Tomás-Barberán et al., 2016; Singh et al., 2017). However, these effects are difficult to assign to polyphenol independent of dietary fiber present in the food matrix (Cuervo et al., 2014). Dietary fat sources like fish oil and lard affect gut microbiota. For example, feeding fish oil-derived lipids to mice resulted in greater abundance of *Actinobacteria*, *Verrucomicrobia* and lactic acid bacteria with concurrent protection from inflammatory and metabolic dysfunction compared to those fed a high-lard diet (Caesar et al., 2015). Fermentation by commensal bacteria like *Clostridia* of plant-derived nutrients leads to the production of short-chain fatty acids like butyrate and propionate. Butyrate is the source of energy for colonial epithelial cells and has anti-inflammatory effect on these cells by through epigenetic mechanisms. Butyrate acetylates FOXP3 (forkhead box P3) promoter and induces the differentiation of T-regulatory cells (Tregs) that helps in reducing intestinal inflammation (Figure 1; Furusawa et al., 2013) and ameliorates IBD. In human clinical studies, administration of prebiotics like FOS and GOS showed a reduced awakening cortisone reaction, a biomarker of anti-anxiety and anti-depression, rats on prebiotics also showed high levels of brain derived neurotrophic factor (BDNF), serotonin receptor 5-HT and low levels of cortisone and L-Tryptophan which suggests that prebiotics can relieve mood disorders (Liu and Zhu, 2018).

#### Probiotics

Probiotics are ingestible viable microorganisms which have garnered much attention as a means to influence the configuration of gut microbiota to provide health benefits for the host (Woloszynek et al., 2016). Once ingested, these bacteria have been shown to promote epithelial barrier integrity, produce antibacterial compounds, ferment indigestible fiber, regulate the acidity of the gut lumen, modulate inflammatory responses, contribute to amino acid and vitamin production, prevent colonization of pathogenic microorganisms, and support maintenance of the gut-brain-axis (Kristensen et al., 2016; Woloszynek et al., 2016). These microorganisms are mainly lactic acid-producing *Lactobacillus* and *Bifidobacterium* strains that are thought to impact the existing microbial structure/function or the host epithelial barrier integrity and immune system regulation (Bermudez-Brito et al., 2012). Furthermore, these bacteria may produce deconjugated bile acids that increase their survival in the gastrointestinal (GI) tract (Begley et al., 2006). Patients with high anxiety levels show increase in sleep time after 3 weeks on probiotics. These patients also showed constitutional change in bacteria: *Lactobacillus* and *Bacteroides* increased, *Clostridium* family (spiral bacteria, *Blautia*) decreased, *Actinomycetes* decreased (Collins bacteria mainly decreased) (Liu and Zhu, 2018). On the contrary, the lack of microbial colonization or inconsistent outcomes with probiotics have previously created difficulties in determining their effects on host health.

For example, a few studies have demonstrated a potential link between probiotic supplementation and GI tract infection, diarrhea secondary to GI infection, and general bouts of persistent diarrhea. Both Goldenberg et al. (2013) and Shen et al. (2017) found beneficial effects of probiotics on improving *Clostridium difficile* infection among children and adults. Similarly, Yang et al. (2017) also determined that subjects (28 randomized control trials, RCTs) with postoperative infections after undergoing GI surgery had fewer complications, shorter hospital stays, and shorter durations of antibiotic use compared to controls on probiotic supplementation. Another meta-analysis (4 RCTs) also concluded that probiotic supplementation may reduce duration of persistent diarrhea in children, but the evidence is limited (Bernaola Aponte et al., 2010). Despite all these studies showing efficacy of probiotics, Allen et al. (2010) advised caution in developing probiotic regimens since their meta-analysis (63 RCTs) found that effect-sizes from study-to-study varied considerably.

The literature linking probiotic supplementation to other diseases is still limited and often far less convincing than the links to GI tract infection and diarrhea. Although only non-high-throughput techniques were used, McFarland reviewed 63 studies and concluded that the evidence is lacking to support a definitive role for probiotic supplementation (McFarland, 2014). When Ghouri et al. (2014) reviewed 35 RCTs for both types of inflammatory bowel disease (IBD) (Crohn’s disease and ulcerative colitis), notably linked to gut dysbiosis (Gevers et al., 2014), their evidence does not support probiotic supplementation for Crohn’s disease, but may be for ulcerative colitis and pouchitis. Their conclusion with respect to ulcerative colitis contradicts that of Naidoo et al. (2011), who performed a meta-analysis of four RCTs, two of which were reviewed in Ghouri et al. (2014), concluding that evidence does not support the efficacy of probiotic supplementation for the maintenance of ulcerative colitis remission. These inconsistencies may have stemmed from the majority of the included studies being limited in sample size, trial dropout rate, and duration, use of histological and endoscopic examinations and presence of placebo groups.

A meta-analysis that included 30 RCTs demonstrated reduced incidence of stage II and III necrotizing enterocolitis, late-onset sepsis, and mortality in necrotizing enterocolitis in preterm infants (Dermyshi et al., 2017). Rees et al. (2017), on the other hand, included 19 RCTs, and despite including many of the studies used in Dermyshi et al. (2017), as well as using the same statistical approach (a random effects regression model), across all RCTs, found no significant associations between probiotic supplementation and the incidence of stage III necrotizing enterocolitis, surgical intervention, and mortality. The disagreement between Dermyshi et al. (2017) and Rees et al. (2017) suggests that the RCT selection criteria is likely critically important when performing a meta-analysis but may be indicative of a larger issue: current RCTs exploring probiotic efficacy are poor in quality. Fiocchi et al. (2015) similarly reported little confidence in the estimated effects to recommend probiotic use for allergy prevention in children from 21 RCTs due to poor quality. Probiotic RCTs are often underpowered, poorly designed (not randomized, controlled and double-blinded, lack *a priori* power calculations, fail to correct for multiple comparisons, etc.), and heterogeneous in terms of age, sex, and demographics (Kristensen et al., 2016). Moreover, the number of studies that even meet the selection criteria to perform a systemic review or meta-analysis is very small, for example, despite a meta-analysis suggesting that a probiotic cocktail of *Bifidobacterium lactis* and lactic acid bacteria can improve GI discomfort, it only included 3 RCTs (Eales et al., 2017).

#### Current Challenges in Studies Using Prebiotics and Probiotics

Research has established various ways in which microbiota coexist with their host, but strong evidence via pre- and probiotic supplementation on health outcomes is still unclear. What currently exists is a large amount of correlative studies, linking host outcomes to supplementation, or studies focused on intermediate outcomes to demonstrate the effects of prebiotics and probiotics on microbiota. The term ‘synbiotics’ has recently been coined to describe a combination of probiotics and prebiotics that promotes the function of probiotics and colonization of beneficial microbes (Gibson et al., 1995). Synbiotics show synergistic effects on the composition of gut microbiota, rather than the probiotic components alone (Saulnier et al., 2008). The very fact that conclusions differ among meta-analyses and systemic reviews, however, should suggest that the evidence is quite variable, and hence it is often difficult to justify prebiotic and/or probiotic supplementation for the majority of disorders, yet probiotics are routinely available and marketed toward disease-free consumers (Scudellari, 2016; Jabr, 2017).

Importantly, there lacks any consensus definition of what constitutes a “healthy microbiome,” so efficacy is not easily inferred by shifts in the configuration of microbiota; little information exists describing how different modes of administration and particular bacterial strains influence health outcomes; and dose-response guidelines only exist for GI disorders (Guarner et al., 2012; Kristensen et al., 2016). Moreover, the proximal effects probiotics have on commensal bacteria do not necessarily tell the entire story as to how probiotics impact host health outcomes (Kristensen et al., 2016); that is, the relationship between host health outcomes and probiotic supplementation is strictly correlative. Given the fact that little evidence suggests that probiotic supplementation impacts the configuration of microbiota in a dysbiotic state, it begs the question of what impact does supplementation have on the configuration of microbiota in disease-free individuals. Kristensen et al. (2016) reviewed studies that assessed differences in the composition of microbiota in healthy adults given probiotics and placebo. Of the seven studies included, only one demonstrated any shifts in composition (specifically, beta diversity), but this study was notable for its crossover design which may have led to carryover effects. No differences in composition were detected between the two groups in any of the other six studies.

Overall, prebiotics and probiotics supplementation for stable changes in the gut microbiome will require further assessment of functional microbial metabolic markers of diet-induced response. In addition, target populations and their background diets need to be considered as confounding variables since dietary components can bias the effect on the gut microbiome (Salazar et al., 2014). Inter-individual variations in response to diet should also be clarified by further characterization of internal environment including metabolic status of responders versus non-responders to dietary changes. Ultimately, addressing these inter-individual differences with a focus on dosing, routes of administration and interaction of specific bacterial strains would help establish the relationship between dietary inputs and health outcomes of the host, and in turn, create individualized recommendations to reduce chronic disease risk.

Even though the gut microbiome studies are contradictory, but the recent invention of the gut-on-a-chip device seems promising in understanding the gut microbiome interactions, the gut-immune pathologies, and perhaps the gut-brain axis. Gut-on-a-chip is a microfluidic human gut device that simulates the gut epithelial barrier function, and host-microbiome interactions. By coupling microbial and immune cells in a spatio-temporal manner, gut-on-a-chip has shown that the intestinal epithelial barrier is quintessential for the healthy function of gut (Shin et al., 2019).

#### Community Modeling and Prediction as a Strategy for Establishing a Healthy State

The concept of community ecology arose in plant and animal ecology (Konopka, 2009) but other theoretical and computational principles have been developed recently in epidemiology and computational social sciences. Organisms that live together in a contiguous environment form a community in which they can interact with each other. A microbial community, discretizeable as a metacommunity (see sections “Host-Environment-Microbiome Interactions” and “Pattern-Oriented Models” for pattern-oriented models aimed to assess assumption-free community interdependence), can be viewed as a group of microorganisms that interact with each other in a microenvironment. From a complex system perspective, a stable microbial community can rest at an equilibrium, which corresponds to a local or global minimum point in the dissipated energy landscape representing all microbiome potential states (see the middle plot of Figure 3B for an example of minimum points in such an energy landscape). When some environmental stressors are input into the system, the composition and function of this microbial community can change in response to the input. Microbial communities show higher-order properties that are not present in individual microbes but arise from their interaction (Song et al., 2014). Thus, the microbial community can be viewed as a complex adaptive system where patterns emerge from global scale interactions vs. individual microbial properties. This is helpful for understanding their behavior and to mathematically model microbial communities. Moreover, mathematical models of these communities provide a way to predict their dynamics as well as control them as a system. These models can be applied to one isolated community, for an ensemble community, or to more communities that are spatially linked (see Figures 2, 3).

The interactions in a microbial community can be on very different spatial and temporal scales that define the structure and function of a microbial network (Li and Convertino, 2018). Habitat structural features are much harder to modify but microbiome function seems highly delicate in particular in response to extreme stressors. For example, biological processes can vary over nine orders of magnitude from an enzymatic reaction to seasonal community succession (Song et al., 2014), leaving aside the spatial dimension where the microbiome is analyzed. Different models have been adopted to capture the interactions on different scales. For example, population-based approaches are often used to model the systemic interaction between species whereas individual-based models are suitable for understanding behavior, in the form of decision rules, of individual cells or other elementary units. Hence, we can classify mathematical models based on the scale of interactions that are aimed to be represented, whether more-focused on macro collective behavior or individual decisions. The modeling choice, however, should ideally not result in dramatically different results for the same pattern. Song et al. (2014) reviewed mathematical models for different interaction units, such as Stoichiometric Model-Based Analysis and Metabolic Function-Based Dynamic Modeling.

With the help of DNA sequencing and metagenomics techniques, we can estimate the abundance of species by analyzing the temporal metagenomics samples. Stein et al. (2013) proposed using generalized Lotka–Volterra (GLV) equations (that are pattern-oriented statistical physics models; see section “Pattern-Oriented Models”) to analyze temporal metagenomics samples and account for time-dependent external perturbations. The GLV equations consists of autonomous, non-linear, coupled first-order ordinary differential equations of the form shown by Eq. 1 (Stein et al., 2013). It has been shown that microbiota temporal dynamics can be predicted with the help of such models, which do have some theoretical ecological foundation, such as a connection to neutral and niche dominant dynamics of the microbiome (Zeng et al., 2015). Gibson et al. also used GLV to model the dynamics of population in microbial communities (Gibson et al., 2016).

(1) $$\frac{d\,x_{i}\,\left(t\right)}{d\,t}=\mu_{i}\,x_{i}\,\left(t\right)+x_{i}\,\left(t\right)\,\sum_{j=1}^{L}M_{i\,j}\,x_{j}\,\left(t\right)+x_{i}\,\left(t\right)\,\sum_{l=1}^{P}\varepsilon_{i\,l}\,u_{l}\,\left(t\right)$$

*x*_*i*_(*t*) is the abundance (or relative abundance) of a species *i*,*i* = 1,⋯,*L*, at time *t*, μ_*i*_ is the model-based (and potentially habitat-dependent) growth rate of species *i*, *M*_*ij*_ is the effect of the interaction of species *j* on species *i* (potentially estimated via pattern-oriented models or assumed to reflect a particular structure) and ε_*il*_ is the susceptibility to the time-dependent perturbation *u*_*l*_(*t*). The first term, μ_*i*_*x*_*i*_(*t*), captures the fluctuation of the species itself (related to the species entropy), the second term, $x_{i}\,\left(t\right)\,\sum_{j=1}^{L}M_{i\,j}\,x_{j}\,\left(t\right)$, describes the interactions between different species (e.g., a proportion of the inferred transfer entropy, such as that described by Li and Convertino (2018) or an estimate based on an empirical assessment), and the third term, $x_{i}\,\left(t\right)\,\sum_{l=1}^{P}\varepsilon_{i\,l}\,u_{l}\,\left(t\right)$, accounts for the influence of perturbations such as an antibiotic or diet or other stressors. The third term, that is a multiplicative noise term, is sometimes considered optional when analyzing stable states of a microbial community without taking one or all perturbations into consideration, in other words, when assuming that the microbiome is not subjected to the environmental fluctuations.

It should be noted that Eq. 1 does not necessarily expresses only a niche-dominated ecological process because diverse species interactions are explicitly accounted for; the model can exhibit a pure neutral model if interactions are balancing out and summing up to zero. Niche processes are much more dependent on environmental dependencies if taken into account in the first term of Eq. 1 or when those affect interaction dynamics (Li and Convertino, 2019). The fixed point or stable state of the system can be determined by setting $\frac{d\,x_{i}\,\left(t\right)}{d\,t}=0$ and solving for *x*_*i*_(*t*). The solutions correspond to the steady state species abundances, which may correspond to either a healthy or unhealthy state; however, stability is often associated with a disease-free condition and this is reflected by the monomodal low energy state in the ecosystem potential landscape (Figure 3B, third plot). When we introduce a new species into a stable community, we can potentially predict the abundance of each species when the system is stable (or, in some cases, unstable) by modeling such event if the microbial interactions are represented correctly; in other words, if the topology of interactions has been correctly inferred which is not an easy task. The accuracy of our models can be confirmed by determining whether the patterns predicted by the model correspond to the observed ones considering the probability distributions of all factors. Inferences can be made by predicting the system response to some certain inputs/perturbations (see Figure 3). For example, Stein et al. (2013) discovered a group of commensal microbes that potentially protect against infection by the pathogen *C. difficile* and proposed a possible mechanism to explain how antibiotics can make the host more susceptible to infection. This type of inferences must first validate the model on baseline conditions and after predict microbiome response under stressors.

With the model being established and the state of system being predicted, we can try to control the system and drive the microbial community to a state we desire (via prebiotics and/or probiotics, for example). The intestinal microbiota is an ecosystem susceptible to external perturbations such as dietary changes and antibiotic therapies (Bucci and Xavier, 2014). Large-scale natural ecosystems, such as coral reef microbiome that share many similar microbes with humans, are subjected to similar stressor patters (e.g., relate to temperature shocks) and interventions (via probiotics). Hence, if we can establish a mathematical model for intestinal microbiota or other ecosystems, then we can associate the resultant species abundance, interactions and biodiversity patterns with perturbation/inputs and engineer microbiome ecology and evolution in both short and long terms. Taking this a step further, it may be possible to drive a subject’s microbial community configuration to one associated with a healthy state by applying perturbations such as dietary changes or antibiotic therapies. Specifically, healthy individuals are known to have certain topologies of microbial community network configurations (Li and Convertino, 2019). The same can be said for large-scale ecosystems where structural habitats can be altered to cause a change in the ecohydrodynamics and microbial functional biodiversity leading to a healthy state. Care should be placed in maximizing functional diversity vs. taxonomic diversity; the latter with unintended consequences and typically associated to dysbiosis due to the presence of invasive species. With a working model of the effects (or outcome) of input changes on the microbial community, we may adjust the microbial community configuration in a controlled manner to match a configuration known to be associated with a healthy state (Figure 1). For example, Gibson et al. (2016) proposed to control the presence and absence of some strongly interacting species to steer the microbial composition to a healthy configuration. Thus, the stratification of healthy individuals based on the relative abundances of their microbes, that correspond to different network topologies of microbal interactions, holds promise for drastically improving personalized medicine (Gibson et al., 2016) informed by microbial population ecology. Equivalently, targeted environmental management can be performed by altering the microbiome supporting habitats or by modifying directly the microbiome network via host introduction (e.g., via introducing corals in reefs) or bacteria inoculation (e.g., via the introduction of microbial mats).

#### Ecohydrological and Engineering Control of the Microbiome

In addition to intervention within a host-microbe system, effective control of the microbiome constituents within an environment can be used to engineer large-scale changes in the environment. Consider, for example, the effects of runoff and water treatment on microbial ecosystems. Engineering control of microbe-related issues is a relatively old practice within the field of water treatment (Pinto et al., 2014). However, these practices are typically designed *ad hoc* to stop the spread of selected microbes of concerns such as coliforms and specific pathogenic organisms rather than targeting the whole microbiome (leaving aside of course whether that is a matter of concern and feasible). What is certainly novel is the control of microbes for enhancing biodiversity and that is still a highly difficult procedures in natural large-scale ecosystems. Current serious problems such as antimicrobial resistance, also related to the widespread diffusion of point source complex mixtures, make any ‘’old” environmental engineering control dated. The massive uses of antibiotics have turned wastewater into an environmental reservoir of antibiotic resistant bacteria (ARB) and antibiotic resistance genes (ARGs). Therefore, without appropriate treatments, wastewater may disseminate antibiotic resistance to various environments, such as soil, groundwater and surface water through seepage and runoff (Wang and Yu, 2012). Eventually, ARB and ARGs enter the food chain through crops grown on the affected land and aquaculture products (Wang and Yu, 2012). Increasing levels of contamination, from antibiotics and antibiotic resistance genes in water bodies and bottom sediments, promote the abundance of drug resistance genes in the microbiota of animals exposed to those water bodies (Amos et al., 2014). Although antibiotic resistance is mostly carried by commensal bacteria, ARGs can also be transferred to pathogens of both animals and humans through LGT (Brody et al., 2008). Wide dissemination of ARGs, via HGT in various microbiomes, adversely impacts the effectiveness of pharmaceutical antimicrobials against infections. Conventional wastewater treatment processes include a combination of physical, chemical and biological approaches to eliminate or reduce suspended solids, organic matter, nutrients and pathogens. However, they are not effective in term of inactivating ARBs and destroy ARGs (Rizzo et al., 2013). Actually, high bacterial abundance and diversity in activated sludge can promote LGT of ARGs (Moura et al., 2012), and this highlights how the ecological paradigms of maximizing taxonomic diversity may not necessarily associate with healthy population outcomes. Some studies showed that biological processes might positively affect ARB strains’ spread and selection as well as ARG transfer (Rizzo et al., 2013) and some other studies reported the slight increase of AMR after conventional wastewater treatment (Ferreira da Silva et al., 2007; Luczkiewicz et al., 2010). Although most bacteria in water can be effectively removed after disinfection, some of them may survive and proliferate due to their resistance to disinfectants (Gerba et al., 2003). Several studies have demonstrated that disinfectants induce transcription of genes encoding virulence and antibiotic resistance by using model bacteria (Chang et al., 2007; Rizzo et al., 2013).

To assess the effectives of engineering controls, it is critical to examine the response of microbiota to them in terms of the dynamics of structures and functions. Moreover, within an ecosystem perspective, these engineering controls should be certainly combined with non-point source management and ecohydrological controls at the river basin scale since water and ecological dynamics shape the microbiome. Harmful microbiota may contaminate runoff due to poorly managed livestock operations, septic systems, the over application of human sewage sludge, contaminated storm sewers, and sanitary sewer overflows (Wiggins et al., 1999). This unhealthy microbiota in the runoff as well as the overload of other compounds such as nitrates propagates their negative effects geographically, along the whole river basins to the ocean, and biologically, from microbes to animals and humans beyond water bodies. Agricultural operations account for a large percentage of all non-point source pollution of ARB and ARGs to the environment due to the widespread use of the veterinary antibiotics (Wiggins et al., 1999). Therefore, managing animal waste to minimize contamination of surface water and ground water is critical. The management of manure collection and storage can minimize runoff and leaking from livestock farm, including the use of vegetative filter, catch basins and clean-water diversion ditches, etc. (Spiehs and Goyal, 2007). Proper biological treatment (e.g., composting, aeration, anaerobic digestion) or chemical disinfection (e.g., chlorine, lime stabilization, UV, ozone) should be conducted to reduce the pathogens and ARB inside before disposal or land application (Spiehs and Goyal, 2007). However, the effectiveness of those management and ecohydrological control approaches needs to be investigated further and likely a portfolio approach of multiple environmental controls is needed (Convertino and Valverde, 2019).

#### Potential for Nanotechnology

Nanoparticles, materials that are from 1 to 100 nanometers (nm) in size, have been used for synthesis of metal nanostructures, nanofibers, nanotubes, nanorods, nanofluids, semiconductors, quantum dots, nanoalloys, and magnetic crystals (Aziz et al., 2015; Prasad et al., 2016; Singhal et al., 2017), sustainable agriculture (Prasad et al., 2017), in dermatological topical applications (Antonio et al., 2014), medicine (Mauricio et al., 2018), and also, more recently, in microbiome interventions, with a particular focus directed toward for cancer treatment and blockade therapy (Karavolos and Holban, 2016; Song et al., 2019). Many metals are used in nanomedicines – for example, silver nanoparticles are used as antimicrobials. *Mucor hiemalis*-derived silver nanoparticles showed significant antimicrobial properties when tested against six pathological bacterial strains like *K. pneumoniae, P. brassicacearum, A. hydrophila, E. coli, B. cereus*, and *S. aureus* along with three fungal pathogens *Candida albicans, Fusarium oxysporum*, and *Aspergillus flavus* (Aziz et al., 2016; Qing et al., 2018). Additionally, silver nanoparticles derived from the fungus *Piriformospora indica* showed increased cytotoxic effects against human breast adenocarcinoma (MCF-7) followed by human cervical carcinoma (HeLa) and human liver hepatocellular carcinoma (HepG2) cell lines as compared to chemically synthesized silver nanoparticles (Aziz et al., 2019). Silver nanoparticles have been also used in wound dressings and coatings for consumer products and biomedical devices (Marassi et al., 2018; Mihai et al., 2019).

Other metals, such as gold nanoparticles are used for imaging, anti-inflammation and infection, titanium dioxide nanoparticles, cerium oxide nanoparticles, zinc oxide, carbon and polymeric, poly (lactic-co-glycolic acid; PLGA) nanoparticles, also have antimicrobial properties (Shaikh et al., 2019). Not only can nanoparticles be used to intervene with the microbiome, but microbial flora can generate metal nanoparticles (Ovais et al., 2018). Nanoparticles can be attached to microbial surfaces especially tumor associated bacteria to improve nanoparticle delivery to tumor site especially in areas of hypoxia containing nitric oxide and reactive oxygen species. For example, *Bacillus coagulans* was used as a factory for nanoparticle synthesis to treat colon cancer (Song et al., 2019). In future nanotechnology has unlimited potential in oral drug therapy, making possible rigorous targeting and controlled drug release in areas of human body where our medicine fails, improvements in the absorption and availability of drugs and gastro-retention for any medical condition, but it is crucial for us to explore microbial interaction with nanoparticles (Mahawar and Prasanna, 2018; Siemer et al., 2018), but engineering a specific nanoparticle to a specific microbe in a specific area remains a challenge (Biteen et al., 2016).

## Conclusion

The significant amount of research on the microbiome in recent years has led to a more-robust understanding of the microbiome and its role in both human health, urban and natural environments. Host-microbe studies, such as investigations of the interactions between the gut microbiome and diet, provide considerable insight into how the microbiome responds to the introduction of new microbes and changes over time and could ultimately serve as a blueprint for interventional and diagnostic techniques based on the microbiome. Similarly, studies of environment-microbe interactions, such as those investigating microbiome diversity within communities, have yielded much information about the role of environmental stimuli in composition and function of the individual microbiome and its evolution over time deepening on environmental structural and functional features.

Models and methods used to evaluate and study the microbiome are critical to developing an accurate understanding of microbiome composition and dynamics. The advent of next generation sequencing and other -omic technologies have made it considerably more efficient to probe the microbiome and generate rich data sets. Moreover, normalization and analysis techniques have been developed to account for the uniqueness of microbiome data collected with these technologies. Some techniques, such as pattern-oriented models and association graphs, have proven to be quite useful in studying microbiome data in association to environmental and engineering controls. Such techniques can provide substantial insight when seeking to identify changes within the microbiome over space, time and biological scales.

Current tools and understanding of the microbiome have enabled researchers to develop new strategies to leverage applications of the microbiome. Analysis techniques have enabled faster and more accurate clinical diagnostics, such as allowing non-invasive sample collection from the surface of skin using adhesives or swabbing in lieu of the invasive biopsies that have been used historically. Moreover, pattern-matching techniques further expand the diagnostic potential of the microbiome to fields as diverse as forensics, where the microbial signature of a sample may provide insight into the source (individual, geolocation, etc.) of the sample. Similar techniques are evolving in ecology and environmental sciences to detect microbial species, hosts and health ecosystem states.

Knowledge of the microbiome also presents potential for interventional strategies, such as personalized medicine or targeted ecological engineering controls in the environment. For example, if we know what the “healthy” state of an individual’s gut microbiome looks like and we know how the gut microbiome responds to external factors, such as diet, we could potentially prescribe a diet for the individual that would restore the gut microbiome from an “unhealthy” state to a “healthy” state. Contamination of public water supplies could be limited using a similar interventional technique. For example, suppose traces of antibiotics are found in a water supply. Evaluation of the microbiome within the water supply and local agricultural sites may help pinpoint the source of contamination, such that appropriate management (disinfection, waste collection, changes in livestock management, etc.) may be put in place at the source before the water supply itself is contaminated. In ecological settings (e.g., coral reefs) alteration of the microbiota via changes of the habitat and/or pro-/pre-biotic treatments can restore biodiversity to its desired levels. Nevertheless, these interventional strategies are still in their infancy and will require additional study before scaling to actual large-scale systematic applications.

As researchers learn more about the microbiome and develop new tools for probing the microbiome, a flood of new questions will continue to arise. For example, the mixed results demonstrated by studies on the effects of prebiotics and probiotics on the microbiome suggest that improved analytical techniques and experimental controls may be needed to gain useful insight into host-microbe interactions. Further, while researchers have made considerable progress in understanding the role of microbial interactions and genetic factors in the evolution of the microbiome, the sheer complexity of the microbiome provides fertile ground for additional studies on distinct microbial communities and inheritance-based studies over generations. Many unanswered difficult questions remain on how healthy natural ecosystems transfer their state to humans in dependence and independently to environmental and population features, and, vice versa how we humans impact natural microbial communities.

Current sequencing technologies also require certain trade-offs between accuracy and speed or resources. For example, WGS can provide a highly accurate picture of the microbes within a sample but requires deeper sampling and considerably more computational resources than marker gene sampling. In contrast, marker gene sampling may require less depth per sample and far fewer computational resources, but it only allows for a determination of relative abundances of microbes and may potentially omit specimens without the marker genes. Consequently, there is a need to develop new methods that provide more-detailed information than marker gene sampling and/or that require fewer resources and less time than WGS, such as the functional annotation techniques described herein. Moreover, there is a pressing need to develop new normalization approaches and analysis techniques (using machine learning and data visualization, for example) appropriate to the idiosyncrasies of microbiome data collected using existing technologies.

As evidenced by the discussion above, the microbiome provides a rich and diverse area of study, which can provide us with diagnostic tools and interventional methods that could ultimately revolutionize medicine, in addition to many other diverse fields such as ecology, environmental sciences and engineering, biotechnology, and computational sciences. Beyond the advancement of research in distinctive fields, microbiome research can also greatly solve grand planetary challenges of humanity such as those related to climate change. To ensure that we can continue probing the limits of the microbiome and developing new strategies to leverage our insights, we need to continue developing robust informatics tools and analytical methods that can process the vast quantities of microbiome data available and guide monitoring and experiments. Such advancements will surely lead to new understandings within this complex field, and it will enable continued growth in microbiome research for decades to come. Moreover, gaining deeper understanding of the microbiome through improved tools and methods will enable engineers and innovators to develop better applications and unlock the potential of the microbiome.

## Author Contributions

CMC organized the authors, assembled and edited the manuscript, and prepared summary and transitional text. KA edited the manuscript and contributed some text to the diet, prebiotics, and probiotics section and brain-gut axes. SB significantly edited the manuscript and contributed to the section on physiological responses to the microbiome. CEC prepared much of the portion related to nutrition, prebiotics, and probiotics. SW provided the portion of the manuscript related to challenges in studies using pre- and probiotics. MC, SM, and YZ prepared the portions related to ecology and the environment-microbe nexus, as well as the portion related to ecohydrological and engineering control of the microbiome. MA and DA-P prepared the portions related to evolution of the microbiome and intra-species microbial diversity and contributed to the microbe and host section. SB, SM, and MA made significant contributions during editing of the manuscript as well. MC, ES, and LK prepared the portions related to properties of microbiome data, statistical analysis, and pattern-oriented models. MC further provided contributions on network inference models. PD provided the portion of the manuscript related to considerations regarding collection strategy. HL provided the portion of the manuscript related to the importance of data normalization. AS provided the portion of the manuscript related to harnessing association graphs to discover co-occurrence relationships. KC and JP prepared the portion related to dermatological diagnostics and tools. ZZ prepared the portion related to community modeling and prediction as a strategy for establishing a healthy state. GR conceived and oversaw the formation of the review, played a central role in bringing the authors together, edited the manuscript, and provided the portion of the manuscript related to microbial forensics.

## Conflict of Interest

The authors declare that the research was conducted in the absence of any commercial or financial relationships that could be construed as a potential conflict of interest.

## Footnotes

1 http://www.earthmicrobiome.org/

2 http://ocean-microbiome.embl.de/companion.html

## Abbreviations

AMP: antimicrobial peptides
AMR: antimicrobial resistance
ARB: antibiotic resistant bacteria
ARG: antibiotic resistance gene
FISH: fluorescence *in situ* hybridization
GI: gastrointestinal
GLV: generalized Lotka–Volterra
GNPS: global natural products social molecular networking
HGT: horizontal gene transfer
IBD: inflammatory bowel disease
ISAPP: International Scientific Association for Probiotics and Prebiotics
LGT: lateral gene transfer
OTU: operational taxonomic unit
PCA: principal components analysis
PcoA: principal coordinates analysis
PCR: polymerase chain reaction
pdf: probability distribution function
qPCR: quantitative PCR
RCT: randomized control trial
RFID: radio frequency identification
RNA: ribonucleic acid
RPKM: reads per kilobase million
rRNA: ribosomal RNA
scRNA-seq: single cell RNA sequencing
TE: transfer entropy
TLR: toll-like receptor
TMAO: trimethylamine-N-oxide
WGS: whole genome sequencing.
